# Specific domains of early parenting, their heritability and differential association with adolescent behavioural and emotional disorders and academic achievement

**DOI:** 10.1007/s00787-019-01449-8

**Published:** 2019-11-30

**Authors:** Iryna Culpin, Marc H. Bornstein, Diane L. Putnick, Hannah Sallis, Ruby Lee, Miguel Cordero, Priya Rajyaguru, Katarzyna Kordas, Tim Cadman, Rebecca M. Pearson

**Affiliations:** 1 Centre for Academic Mental Health, Population Health Sciences, Bristol Medical School, University of Bristol, Oakfield House, Bristol, BS8 2BN UK; 2 Eunice Kennedy Shriver National Institute of Child Health and Human Development, National Institutes of Health, Bethesda, USA; 3 Institute for Fiscal Studies, London, UK; 4 MRC Integrative Epidemiology Unit, The University of Bristol, Bristol, UK; 5 UK Centre for Tobacco and Alcohol Studies, School of Psychological Science, University of Bristol, Bristol, UK; 6 Avon Longitudinal Study of Parents and Children (ALSPAC), Bristol Medical School, University of Bristol, Bristol, UK; 7 Department of Epidemiology and Environmental Health, School of Public Health and Health Professions, University At Buffalo, Buffalo, USA; 8 NIHR Biomedical Research Centre, University of Bristol, Bristol, UK

**Keywords:** Avon Longitudinal Study of Parents and Children (ALSPAC), Parenting, Adolescence, Behavioural disorders, Academic achievement, Specificity

## Abstract

Variations in parenting across large populations have rarely been described. It also remains unclear which specific domains of parenting are important for which specific offspring developmental outcomes. This study describes different domains of early parenting behaviours and their genetic heritability, then determines the extent to which specific domains of parenting are associated with later offspring outcomes. Parenting behaviours (birth to 3 years) were extracted from self-reported questionnaires administered to 12,358 mothers from the UK-based birth cohort study, the Avon Longitudinal Study of Parents and Children and modelled as a latent factor using Confirmatory Factor Analysis. Genetic heritability and correlations between parenting factors were estimated using genome-wide complex trait analysis. Three parenting factors were derived: parental enjoyment, conflictual relationships and stimulation; all showed low genetic heritability. There was no evidence of association between parental enjoyment and offspring behavioural disorders and depressed mood. Stimulation was associated with better English grades (standardised *β* = 0.195, *p* < 0.001) and enjoyment was negatively associated with English grades (*β* = − 0.244, *p* =  < 0.001). Conflictual relationships were associated with higher risk of offspring behavioural disorders (*β* = 0.228, *p* = 0.010) and depressed mood (*β* = 0.077, *p* = 0.005). Higher enjoyment reduced the association between conflict and behavioural problems (interaction term *β* = 0.113, *p* < 0.001). We found evidence for predictive specificity of early parenting domains for offspring outcomes in adolescence. Early stimulation, unlike enjoyment, promoted later educational achievement. Conflictual relationships were associated with greater risk of behavioural problems, buffered by increased enjoyment. These findings hold implications for parenting interventions, guiding their focus according to the specificity of parenting domains and their long-term outcomes in children.

**Electronic supplementary material:**

The online version of this article (10.1007/s00787-019-01449-8) contains supplementary material, which is available to authorized users.

## Introduction

Variations in mother–child interactions and the quality of early parenting are associated with long-term child outcomes, including mental and physical health, socioemotional and cognitive developments [1, 2]. However, parenting is a complex construct. Parents not only nurture and protect children, they also guide them in understanding and expressing appropriate feelings and emotions as well as educate and prepare them for adaptation to a wider range of life roles and contexts [1, 3]. Parents also deal with disciplining and conflicts as their children grow and take risks [4, 5]. Thus, parenting practices constitute a varied and demanding set of skills and there is considerable variation in how adults esteem and execute different components of the caregiving repertoire. That said, different aspects of the parenting experience across large populations of parents have not yet been adequately described [6–8].

### Parenting and child outcomes

Apart from describing variations in parenting behaviours, it is important to understand the long-term impact different parenting domains have on child outcomes. Parenting practices are often cast along three main domains, warmth/love or enjoyment, control/discipline/conflict and stimulation [1]. Warmth encompasses enjoyment, sensitivity and involvement [9], with higher levels of warm, sensitive and developmentally stimulating parenting being associated with decreased child negative reactivity [10] and greater academic achievement [11]. By contrast, parent–child relationships characterized by relatively low levels of warmth and affective enjoyment are associated with offspring emotional [12] and behavioural [13] problems in adolescence. Parental control encompasses monitoring and harsh discipline [14], with higher levels of conflict within the parent–child relationship and harsh discipline being associated with offspring behavioural problems in adolescence [15]. Stimulation, defined as parental activities to promote learning [16], has been found to predict offspring cognitive abilities [17] and academic achievement [18].

### Importance of specificity

Although links between parenting and child outcomes are well documented [1, 2, 19], the importance of specific aspects of parenting for particular child outcomes has rarely been studied. Parenting interventions can be complex and taking a ‘one size fits all’ approach is often ineffective, with many universal efforts failing to show evidence of positive effects across all outcomes (Triple P-Positive Parenting Program; The Nurse-Family Partnership) [20, 21]. Thus, it is essential to establish links between specific parenting domains and specific child outcomes to design targeted parenting interventions. Here, we examine the extent to which specific parenting domains are associated with specific offspring outcomes. We hypothesise that enjoyment/warmth will be associated with emotional, conflict with behavioural and stimulation with academic offspring outcomes.

### Genetic basis of different domains of parenting

Variations in different components of parenting will be driven by both genetic and environmental factors [6]. Associations between parenting and child outcomes could be explained by shared genetic liabilities. The extent to which this may be the case will primarily depend on how heritable parenting is, with genetic confounding playing a greater role if these parenting traits are more heritable. Thus, it is important to estimate heritability [(*h*^2^): the proportion of variation that can be attributed to genetic differences for the particular context and time-point] of the specific parenting traits investigated. Meta-analysis of previous research based on twin and adoptive studies indicates a moderate genetic basis (23–40%) across most parenting phenotypes [22], with some evidence for variation in genetic influence depending on the parenting components measured. For instance, parental genetic factors explained less of the ‘negative’ aspects of parenting such as conflict with children than the ‘positive’ aspects such as warmth and enjoyment [22]. However, these studies have used twin designs to estimate *h*^2^, which often over estimate heritability [23]. An alternative approach is to use molecular genetic data and estimate the heritability captured by single nucleotide polymorphisms (SNPs) included in genotyping platforms [24]. This approach has not previously been applied to the heritability of parenting. Here, we describe different components of parenting experiences and estimate SNP-based *h*^2^ from maternal molecular genetic data to indicate the potential role of genetic confounding.

### Current study

In the current study, we address the limitations of previous research by describing the different domains of self-reported parenting in the first 3 years of life and estimating the extent to which these domains are associated with emotional and behavioural disorders and academic achievement in offspring at age 16 years using data from a large UK-based birth cohort study, the Avon Longitudinal Study of Parents and Children (ALSPAC). The unique richness of the ALSPAC data provides a rare opportunity to control for early measures of child behavioural problems that may affect parenting, thus, controlling for reverse causality. We also utilise molecular genetic data to estimate the variance explained by genetic factors and to examine shared genetic architecture across factors.

## Methods

### Participants and procedure

The sample comprised participants from the Avon Longitudinal Study of Parents and Children (ALSPAC). During Phase I enrolment, 14,541 pregnant mothers residing in the former Avon Health Authority in southwest England with expected dates of delivery between 1 April 1991 and 31 December 1992 were recruited. When the oldest children were approximately 7 years of age, an attempt was made to bolster the initial sample with eligible cases who had failed to join the study originally. The total sample size for analyses using data after the age of 7 years is 15,247 pregnancies, of which 14,899 were alive at 1 year of age. Our sample comprised 12,358 mothers with at least one parenting item. Ethical approval and informed consent for the study was obtained from the ALSPAC ethics and law committee and the local research ethics committees. Informed consent for the use of data collected via questionnaires was obtained from participants following the recommendations of the ALSPAC ethics and law committee at the time. Detailed information about the cohort has been collected since early pregnancy, including regular self-completion questionnaires from mothers and children. Information about ALSPAC is available at www.bristol.ac.uk/alspac/, including a searchable data dictionary (https://www.bris.ac.uk/alspac/researchers/data-access/data-dictionary/). Further details on the cohort profile, representativeness and phases of recruitment are described in two cohort profile papers [25, 26].

## Measures

### Development of Parenting Factors

#### Process of item selection

Full details of item section and development of parenting factors are provided in Supplementary Material. In summary, potential items were extracted from self-reported questionnaires administered from pregnancy to age 3 years capturing parenting behaviour, attitudes and knowledge. Items categorised as parental enjoyment, conflictual relationships, and stimulation and teaching (based on parenting taxonomies) [27] were extracted and entered into separate single-factor confirmatory factor analysis (CFA) models. We focused on ages 0–3 years to capture a period of time most mothers spend with their children prior to the commencement of nursery school.

### Adolescent outcomes

#### Depressed mood

The short mood and feelings questionnaire (SMFQ) [28] was administered at age 16 years via postal questionnaires. It consists of 13 items relating to low mood during the past 2 weeks, each with scores of 0–2. Individual items are summed, producing a total score ranging from 0 to 26, which was dichotomised to classify individuals as depressed versus not-depressed using a cut-off point of ≥ 11. This cut-off point has been shown to have high sensitivity and specificity [29] and has been applied in previous studies based on community samples [30].

#### Behavioural disorders

Behavioural disorders were assessed using parent and child versions of the Development and well-being assessment (DAWBA) [31]. The semi-structured interview comprises questions about a range of symptoms relevant to childhood psychiatric disorders. At age 15 years, the parent-completed DAWBA was used to assess symptoms of disruptive behaviour disorder (DBD), oppositional defiant disorder (ODD) and attention deficit hyperactivity disorder (ADHD) over the past 6 months or conduct disorder over the past year. Children are not asked in detail about behavioural disorders due to possible bias in reporting these conditions [32]. Child-reported versions of the DAWBA were used to assess symptoms of major depressive disorder (MDD) and generalised anxiety disorder (GAD) over the past 6 months. Binary variables were derived to characterise diagnosis of emotional and behavioural disorders (versus no diagnosis).

#### Educational achievement

General certificate of secondary education (GCSE) grades achieved in English language at age 16 years were extracted from external educational records and with consent linked to ALSPAC identification numbers. A binary variable was created to represent either failing to achieve an A*–C in English (coded as 1) or achieving A*–C (coded as 0), which is an essential qualification in the UK. The same procedure was conducted for deriving GCES in Maths. We focused on the GCSE grades in English to avoid multiple comparisons with a number of GCSE grades. In addition, grades in English are the most relevant outcome to the parenting domain of stimulation and teaching (mostly composed of language-related items: e.g., story-telling, song-singing). However, to avoid reliance on one exam grade only, Maths GCSE was also included in the model as a secondary educational outcome. Results are primarily reported for English (adjusted for Maths), with estimates for Maths provided in the Supplementary Material.

#### Confounding variables

Parental and child characteristics identified in previous studies as being associated with parenting and child outcomes were accounted for in the model. These included highest maternal educational attainment (minimal education or none, compulsory secondary level (up to age 16 years), non-compulsory secondary level (up to age 18 years) versus university level education), maternal antenatal depression (18 and 32 weeks’ gestation) were assessed using the Edinburgh Postnatal Depression Scale (EPDS) [33], maternal age (in years), child gender (male *versus* female) and early behavioural problems were assessed at age 3 years through maternal reports using the total problems scale of the Rutter revised preschool questionnaire [34].

### Analytic strategy

Models were estimated using structural equation modelling (SEM) in M*plus* v.7 [35]. Full information maximum likelihood (FIML) [36] estimator was used to account for the missing data. FIML renders unbiased and more efficient estimates under missing-at-random (MAR) missing data conditions [37]. A model was considered to have a good fit if the root mean square error of approximation (RMSEA) was ≤ 0.06 and the comparative fit index (CFI) and Tucker–Lewis index (TLI) cut-off values were close to 0.95 [38]. The Chi-square test of overall fit is sensitive to model misspecification in instances when sample size is large [39], thus, we gave greater weight to the incremental fit indices [38].

#### Latent factor model

**Fig. 1:**
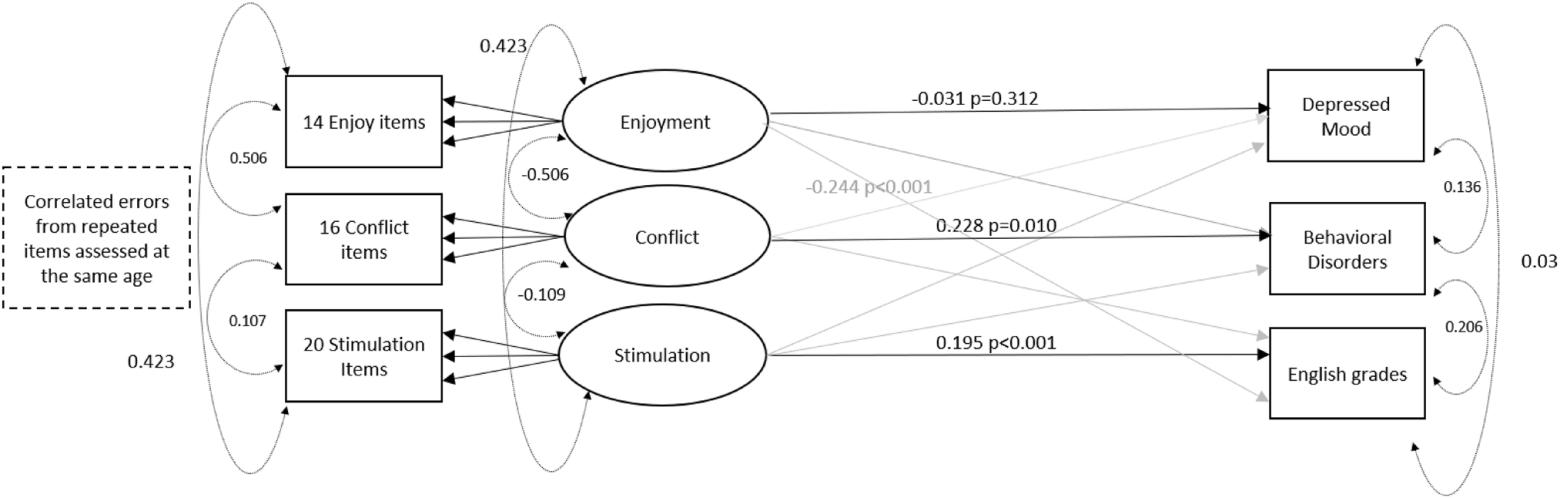
Latent factor model representing associations between parenting factors and adolescent outcomes following adjustments for parental (maternal age, educational attainment, prenatal depression) and child (gender and early child behavioural problems) confounders and accounting for GCSE in Maths. Analyses conducted on all available data for each estimate using WLSMV defaults in M*plus* (*n* = 12,358). Straight arrows represent regression paths, whilst curved arrows represent correlations. The hypothesised and standardised path coefficients are depicted in black, whilst other significant path coefficients are depicted in grey

Full details of latent factor model derivation, including the flow chart of items included into the CFA (Fig. 1S) and derived factors and factor loadings (Table S1), are presented in the Supplementary Material. In brief, items that were both theoretically assigned and showed standardised loadings > 0.15 on the relevant dimension were entered into a combined model using confirmatory factor analyses (CFA) with a robust weighted least square (WLSMV) estimator with covariates and similar assumptions to FIML, to model categorical data [40].

#### Estimating heritability of each of the three parenting factors and genetic correlation between parenting factors

Analyses to estimate heritability and genetic correlations are described in more detail in the Supplementary Material. In summary, we first calculated estimates of SNP-based heritability (*h*^2^_SNP_) for each parenting factor using the restricted maximum likelihood (REML) method implemented within the genome-wide complex trait analysis (GCTA) software [24]. Second, we used a bivariate REML approach to estimate the genetic correlation between each parenting factor with each other to investigate shared genetic architecture across parenting factors. Any overlap here could be due to pleiotropy (genetic effects on multiple traits), shared biological mechanisms between domains or a causal relation from one domain to another.

## Results

Associations between parenting factors and child and parental confounders are presented in Table S2, Supplementary Material. Characteristics of the sample by the completeness of data are presented in Table S3, Supplementary Material.

### Final latent parenting factors

A model using CFA to fit the following three factors with the corresponding items below showed good model fit. The RMSEA (0.024, 95% CI 0.024–0.025) and the CFI (0.92) indicated that the measurement model fits the data well, supporting the adequacy of the model for tests of structural paths. There were relatively high correlations between parenting factors (Fig. 1), however, a second-order or bi-factor model did not converge.

#### Factor 1: Parental Enjoyment

Parental enjoyment contains 14 items relating to enjoyment of the child from ages 4 weeks to 3 years 11 months (e.g., ‘I really enjoy my baby’, ‘Having a baby has made me feel more fulfilled’) as well as items relating to frequency of cuddling and playing with the child. Initially, items relating to feelings of irritation with the child (e.g., ‘This child gets on my nerves’) were included, however, in the final model, they loaded better on the factor encapsulating conflictual mother–child relationship. At age 8 months, majority of mothers reported enjoying their baby (72%) and taking great pleasure in watching their baby develop (93%), whilst at age 1 year 9 months, 96% reported to really love their child. At age 2 years 9 months, 49% of mothers reported feeling more fulfilled by having the child, with an overwhelming majority of mothers expressing physical affection (e.g., cuddling) to their children (98%) at age 3 years 11 months. The internal consistency of parental enjoyment is *α* = 0.82, with the summed items forming a normally distributed scale. Higher factor scores represent less parental enjoyment.

#### Factor 2: Conflictual relationships

Conflictual relationships contains 16 items relating to conflict, harsh discipline and irritation with the child (e.g., frequency of arguments, ‘battle of wills’, smacking and shouting) from ages 1 year 6 months to 3 years 11 months. At age 1 year 6 months, a substantial proportion of mothers reported having battles of wills (37%) and frequent conflict (21%) with their children. In addition, 24% of mothers reported having smacked their children sometimes during tantrums, whilst 58% of mothers reported having shouted at their child. At age 3 years 11 months, 17% of mothers reported that the child gets on their nerves. The internal consistency of conflictual relationships is *α* = 0.75. Higher factor scores signify more conflictual relationships.

#### Factor 3: Stimulation and teaching

Stimulation and teaching contains 20 items relating to the frequency of engagement in teaching and stimulating activities from ages 6 months to 3 years 6 months (e.g., naming parts of the body, colours, numbers, singing to and talking with the child). At age 6 months, a substantial proportion of mothers reported often teaching (37%) and talking (30%) to their child, whilst 62% reported always talking to their child when doing household activities. At age 1 year 6 months, a majority of mothers reported that they teach their child the alphabet (70%), but not songs (7%) or politeness (4%). At age 2 years, 83% of mothers reported that they take their child to the park or playground at least once a week. The internal consistency for stimulation and teaching is *α* = 0.75. Higher factor scores represent less stimulation and teaching.

### Associations between parenting factors and offspring behavioural disorders, depressive symptoms and academic achievement at 16 years

Latent parenting factors were regressed onto offspring depressive symptoms, behavioural disorders and academic achievement at age 16 years in the same model, leading to mutually adjusted associations between each parenting factor and each adolescent outcome. The model was adjusted for a number of possible parental (maternal age, educational attainment, depression) and child (gender and early behavioural problems) confounders. Given the complexity of the model, interaction terms were derived from saved factor scores for each latent factor to investigate interactions between parenting factors. The three interaction terms between continuous scores (stimulation*enjoyment; stimulation*conflict and conflict*enjoyment) were regressed onto each of the outcomes, with parenting factor scores also entered into the model.

Standardised path coefficients (*β*) of the associations between parenting factors and offspring emotional, behavioural and academic outcomes are presented in Table S4, Supplementary Material. There was no evidence of association between parental enjoyment and offspring behavioural disorders and depressed mood at age 16 years. There was evidence for a strong association between parent–child conflictual relationships, offspring behavioural disorders, academic achievement and a smaller association with depressed mood at age 16 years. The longitudinal association with behavioural problems remained independent following adjustment for parental reports of behavioural problems at age 3 years (*β* = 0.227, *p* = 0.007, 95% CI 0.062–0.391). There was a strong association between parent–child conflictual relationships and early childhood behavioural problems at age 3 years (0.681, *p* < 0.001, 95% CI 0.661–0.701), however, there was no evidence for an independent association between early childhood behavioural problems and adolescent behavioural disorders in the mutually adjusted model (*β* = 0.020, *p* = 0.708, 95% CI − 0.087–0.128). There was also evidence for an interaction between conflictual relationships and enjoyment with offspring behavioural disorders at age 16 years (interaction term *β* = 0.113, *p* < 0.001; Fig. S2, Supplementary Material, represents percentage of offspring with CD or ODD diagnosis according to patterns of parental conflict and enjoyment).

Early stimulation and teaching activities were associated with better GCSE grades in English language at age 16 years after controlling for maternal education (*β* = 0.195, *p* < 0.007, 95% CI 0.154–0.236); there was an association of similar magnitude for GCSE in Maths (see, Supplementary Material). There was evidence for a negative association between parental enjoyment and English grades, independent of stimulation and teaching (*β* = − 0.244, *p* < 0.001, 95% CI − 0. 295 to − 0.193). However, there was no evidence for an interaction between parental enjoyment and stimulation. Results were comparable when using complete case sample on all variables (*n* = 2,694; Results S1, Supplementary Material).

### Heritability estimates of and genetic correlations between parenting factors

Estimates of *h*^2^_SNP_ were estimated for each parenting factor for all mothers with genetic information and a parenting score (*n* = 6453). Although effect sizes were small for each factor (suggesting that only a small proportion of the variation in each phenotype is due to genetic variation), larger estimates were observed for stimulation (*h*^2^_SN_ *p* = 0.096, se = 0.05) than conflictual relationships (*h*^2^_SN_ *p* = 0.044, se = 0.05) and enjoyment (*h*^2^_SN_ *p* = 0.040, se = 0.05), although the wide confidence intervals include larger and smaller estimates which overlap. However, these analyses were underpowered and estimates should be interpreted with caution.

Estimates of genetic correlation suggested that the SNP effects for conflictual relationships (*r*_G _= 0.35, se = 0.67) and stimulation (*r*_G _= 0.06, se = 0.60) with low enjoyment act in the same direction, while SNP effects between conflictual relationships and stimulation are negative (*r*_G _= − 1.00, se = 0.99). However, the confidence intervals for each of these relationships are wide and overlapping and as such could include estimates in both directions.

## Discussion

The current study describes three different domains of self-reported early parenting behaviour, estimates the extent to which these parenting domains are heritable and provides longitudinal evidence that links specific domains of parenting with specific offspring outcomes in adolescence, whilst estimating the proportion of variation in these domains that may be attributed to genetic factors. Estimates of heritability for each factor were small. Given the small sample size, the confidence intervals include both larger and smaller estimates, thus these findings should be interpreted with caution.

Our findings indicate that parent–child conflictual relationships in the first 3 years of life are associated with offspring behavioural disorders and depressed mood at age 16 years. This finding is consistent with previous research implicating harsh parental discipline in offspring behavioural and emotional problems [15, 41]. This effect may be partly explained by reverse causality, whereby children who exhibit difficult behaviour contribute to a conflictual parent–child relationship. Indeed, a strong association emerged between parent–child conflictual relationships and child conduct problems at age 3 years. However, the longitudinal association between conflictual relationships in the parent–child dyad and adolescent behavioural disorders remained after adjustment for early childhood behavioural problems. There was no association between early childhood behavioural problems and adolescent behavioural disorders, suggesting that a conflictual parent–child relationship may be a good independent predictor of conduct disorders in adolescence in addition to early behavioural problems.

We found an interaction between conflictual parent–child relationships and enjoyment with offspring behavioural disorders at age 16 years, suggesting a possible ‘buffering’ effect of high parental enjoyment on the negative effect of conflictual and harsh parenting and associated behavioural disorders in adolescence. The mechanisms that underlie such ‘buffering’ by enjoyment remain unclear. It may be that the type of conflict encountered by parents and children reporting both high conflict and enjoyment is different from those who experience conflictual relationships without enjoying the other areas of the relationship. For instance, mothers who report high levels of conflict and enjoyment may be more emotionally expressive and have conflicts that although frequent, are more quickly resolved. High levels of enjoyment may also facilitate a positive emotional environment, where arguments and conflict are regularly resolved and parents and children share positive feelings that further enhance positive parenting and optimal child development [42].

There was an association between conflictual parent–child relationships and depressed mood at age 16 years. However, we found no evidence for an independent association between parental enjoyment, or its interaction, and offspring behavioural disorders and depressive mood at age 16 years. This is not to say that early parental enjoyment and warmth are inconsequential for adolescent behavioural and emotional development; rather, this factor may not capture particular aspects of parenting related to offspring emotions and behaviour. For instance, it has been suggested that parental emotional scaffolding and regulation specifically in response to distress, as well as emotional availability, may be important for child’s emotional and behavioural functioning [43]. This domain was not specifically captured here and rather the enjoyment factor was more related to positive emotion and fun rather than distress.

Unsurprisingly, early stimulation activities and teaching (e.g., reading, story-telling) were associated with better GCSE grades in English language at the age 16 years. Conversely, parental enjoyment was negatively associated with English grades at the end of school. The lack of interaction between parental enjoyment and stimulation suggests that, even in the context of high stimulation, enjoyment is still negatively associated with offspring academic achievement. A parent’s focus on low demandingness and letting children simply enjoy themselves, rather than enforcing learning [44], may eventuate in lower achievement. It should be noted, however, that our findings point to the importance of enjoyment for other offspring outcomes such as its possible protective role in the association between conflictual parent–child relationships and adolescent behavioural disorders.

The strengths of this study include the large sample size, the long-term follow-up, the availability of repeated measures on parenting behaviour across early childhood as well as rich data on confounders and a longitudinal design that enabled examination of associations between early parenting and offspring emotional, behavioural, and academic adjustment in adolescence, whilst accounting for early measures of child behavioural problems to rule out reverse causality. Although it is likely that genetic analyses were underpowered, we were able to utilize molecular genetic data to estimate the proportion of variation in the parenting domains that could be attributed to genetic factors, thus estimating the potential role of genetic confounding. The proportion of variance explained by genetic variants was very low, albeit underpowered, as precision was low, and although inconclusive, it provides an indication that the role of genetic variance is unlikely to explain the results.

The findings need to be interpreted in light of several limitations. First, despite the population-based study design, it was impossible to rule out selection bias in relation to baseline recruitment or attrition in the sample over time. We attempted to address this concern by controlling factors known to predict attrition in ALSPAC (e.g., parental education and psychopathology) and using FIML estimator in M*plus* to account for missing data [36]. Second, we relied on parental reports of parenting behaviour, which may be subject to measurement error. However, measurement error is found in all measures of behaviour, including self-report and directly observed measures [45]. Arguably, for the dimensions of parenting under investigation such as harsh discipline (relatively rare event) and internal feelings of love or irritation, parental report may be an appropriate measure [46], even though it is likely to be affected by social desirability bias. Direct observations of parent–child interactions may not capture such events and are difficult to collect in large population-based samples, whilst it is not possible to collect child-reported parenting between birth and age 3 years. In the present study, however, parenting factors were modelled using a latent variables approach, which explicitly accounts for measurement error by only modelling variance, which is shared across items and separating this from specific variance likely reflecting error [47].

Given our findings regarding possible ‘buffering’ effect of high parental enjoyment on the negative effect of conflictual and harsh parenting and associated behavioural disorders in adolescence, future research is warranted to examine possible mechanisms underlying this interplay. Similarly, future research is needed to provide further insights into the role that conflictual parent–child relationships, parental enjoyment, teaching and stimulation play in adolescent mental health as well as the genetic basis of parenting. Future research may also focus on observing parent–child interactions characterised by self-reports of high enjoyment and conflict and utilising microcoding methodology to uncover the type of behavioural and emotional responses that are associated with enjoyment and have the potential to change the meaning of the conflict. Research utilising genetic data to highlight potential evocative genetic effects, where child genetic risk score is associated with certain responses and future mental health may also enhance this area of research.

## Conclusions

This study shows that different domains of parenting are important for different offspring outcomes. Early conflictual parent–child relationships in the context of low parental enjoyment are a strong predictor of offspring behavioural disorders, whilst early stimulation and teaching are important for subsequent academic achievement in English. This finding implies that strategies to reduce conflicts, but also increase parental enjoyment of the child may be one avenue to reduce the risk of later offspring behavioural problems in families with conflictual parent–child relationships. Furthermore, encouraging early stimulation activities, such as more frequent reading and learning of simple concepts (e.g., alphabet), is likely promote subsequent offspring academic achievement. Future research is warranted to replicate these long-term findings and apply them judiciously in tailored interventions.

## Electronic supplementary material

Below is the link to the electronic supplementary material.

Supplementary file1 (DOCX 125 kb)

## Abbreviations

ADHD: Attention deficit hyperactivity disorder
DBD: Disruptive behaviour disorder
GAD: Generalised anxiety disorder
CD: Conduct disorder
ODD: Oppositional defiant disorder
MDD: Major depressive disorder
GCSE: General certificate of secondary education
